# Key factors and tactical variations in Chinese national women's softball games: a machine learning-based identification

**DOI:** 10.3389/fspor.2025.1701387

**Published:** 2025-11-21

**Authors:** Hanyao Li, Gang Cheng, Tianfeng Zhang

**Affiliations:** 1 School of Physical Education, Nanjing Tech. University, Nanjing, China; 2 Sports Education and Training Science, Beijing Sport University, Beijing, China

**Keywords:** softball, prediction of victory or defeat, key factors, machine learning, athletic performance analysis

## Abstract

**Objective:**

This study employs machine learning to analyze data from Chinese women's softball games, identifying key factors determining game outcomes. It explores patterns in how different teams develop winning strategies.

**Method:**

This study analyzed data from 81 of 296 games conducted between 2023 and 2024, using game outcomes (win = 1, loss = 0) as the target variable and 98 features as inputs. Machine learning models, including Random Forest (RF), XGBoost, KNN, and SVM, were implemented in Python and trained on a 7:3 train-test split. Model performance was evaluated using AUC, F1-score, accuracy, precision, and recall to identify the best-performing model. SHAP and PDP were then employed to evaluate feature contributions to game outcome predictions.

**Results:**

The RF model achieved the highest accuracy on the test set with an AUC of 97.7% (95% CI: 0.938, 0.993). We identified the ten features that had the most significant impact on game results, including P-ER, OBP, RBI, and AVG. PDP analysis further revealed that an increase in P-ER and P-H significantly increased the probability of losing; improvements in OBP and AVG substantially increased the chances of winning. Different teams exhibited varying strategic emphases in their decisive factors: Team SC relied heavily on pitching performance, while SH, LN, and JS prioritized batting strategies.

**Conclusion:**

Feature importance analysis from the RF model indicates that P-ER and key batting metrics (e.g., OBP, AVG)are significantly associated with predicting game outcomes. These findings highlight their importance in predictive models, though further research is needed to confirm their practical impact.

## 1 Introduction

Data have long been fundamental to sports science. During the early days of professional sports, “sports data” served as a valuable tool for answering various questions in the discipline (1, 2). Currently, data pattern analysis has emerged as a leading approach in sports science research. For example, data analysis and data mining techniques are frequently employed in studies of professional sports, such as ice hockey, soccer, and basketball (3–5). Baseball and softball are gaining popularity worldwide as sports integrating technical skills, strategy, and teamwork. With the rapid development of artificial intelligence (AI) technologies, studies increasingly utilize AI-driven analysis of baseball and softball data to optimize tactics, evaluate player performance, and manage injuries (6–10). These data-driven approaches have brought new momentum to the advancement of professional sports. Moreover, they offer more reliable and credible theoretical foundations than traditional statistical methods for predicting game outcomes and determining decisive factors.

Game outcomes have always been a central focus in competitive sports analysis (11–13). Traditional methods, relying on statistical regression and linear analysis (13, 14), often overlook complex nonlinear relationships in game data, limiting their potential in guiding competitions. With the advancement of AI technology, analysis of the intricate nonlinear relationships between game processes and outcomes has become increasingly feasible. Consequently, leveraging advanced machine learning algorithms to uncover hidden patterns in sports competition data has become an important interdisciplinary research direction in sports science (8, 15, 16). Baseball performance analysis frameworks are well-established (17–19), with abundant studies on key factors (19, 20), while softball research remains nascent, with limited depth and scope. However, given the strong similarities between baseball and softball in terms of technical execution, game rules, and tactical systems (21), existing findings from baseball research can provide valuable theoretical and technical insights for the study and analysis of softball data.

This exploratory study investigates the key factors influencing game outcomes in softball matches between evenly matched teams. We hypothesize that interpretable machine learning can capture nonlinear relationships among pitching, defensive, and batting variables and match outcomes. The primary aim of this study is to identify the critical determinants of game outcomes by modeling nonlinear relationships among key game metrics using machine learning algorithms. The secondary aims are to employ explainable AI techniques such as SHAP and PDP to interpret model outputs and reveal tactical and performance variations among teams, and to compare multiple predictive models to determine the optimal balance between predictive accuracy and interpretability.

## 2 Sample and method

**Figure 1:**
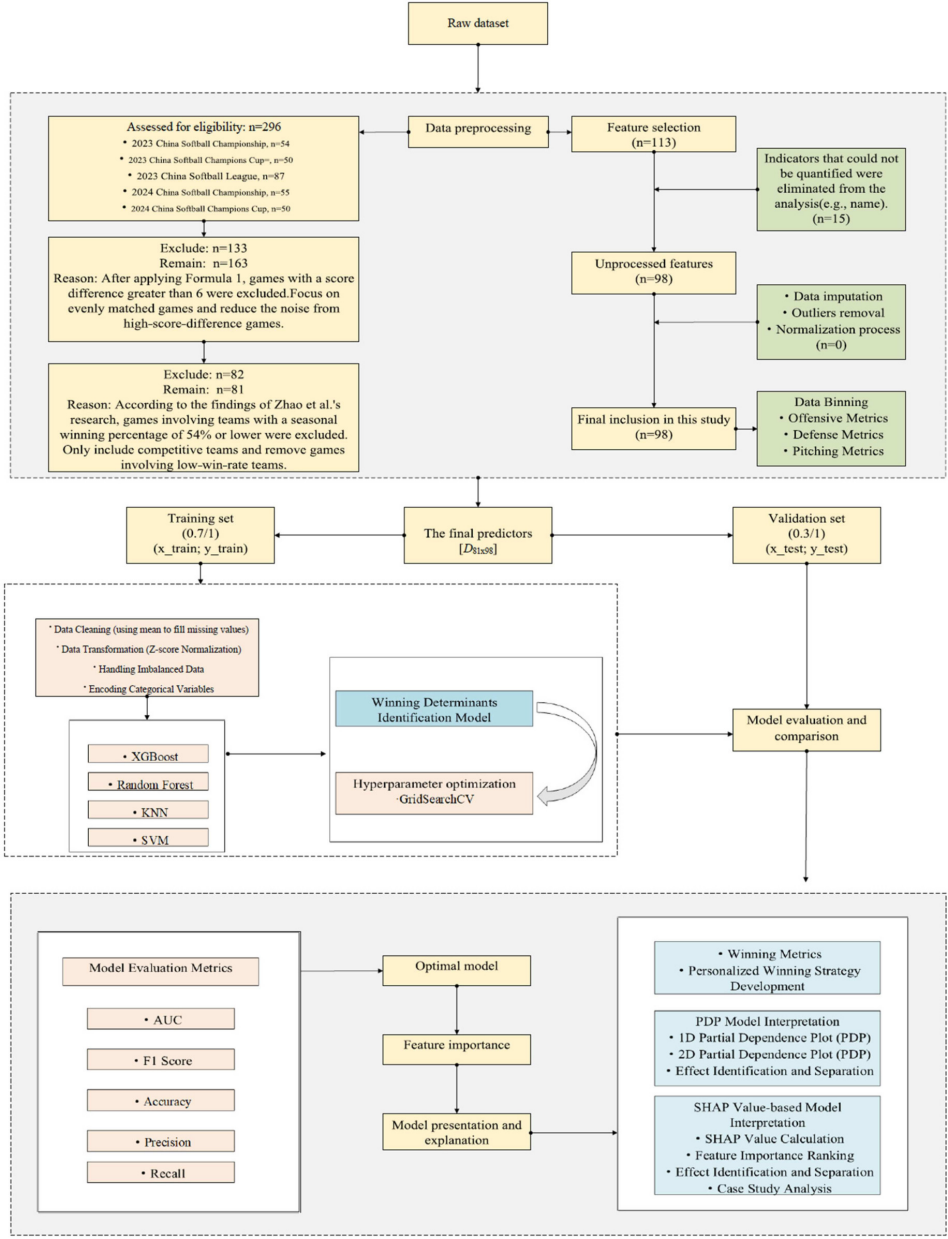
The technical workflow of this study.

The study design is shown in Figure 1.

### 2.1 Research sample

ScorePAD software was used to collect game statistics from the 2023 to 2024 National Women's Softball Championship, National Women's Softball League, and National Women's Softball Tournament. Data from 296 games across five competition stages were initially gathered. However, as games with significant skill disparities between teams offer limited competitive value, data cleaning was performed to remove the data of such games. A score threshold (*T*) was applied for data filtering, calculated using (Equation 1):

$$T=\frac{\sum_{i=1}^{n}X_{i}}{n}+2\cdot \sqrt{\frac{\sum_{i=1}^{n}\left(X_{i}-\bar{X}\right)}{n}}-2$$ (1)

where *n* = 296, $X_{i}$ represents the score difference in game *i*, and $\bar{X}$ is the average score of all games.

Based on the results of (Equation 1) and the distribution of score differences across all games, the score threshold (*T*) was set to 6, corresponding approximately to the 55th percentile of the observed score differences. Games with a score difference exceeding this threshold were excluded to maintain data quality and ensure that only highly competitive matches were analyzed. In addition, a win rate threshold was applied: only games in which both teams had a win rate above 54% during that competition stage were included (13). These criteria ensured that the final dataset comprised high-level matchups between closely matched teams. Through these data preprocessing strategies, 81 games involving four teams—SiChuan (abbreviate as SC), ShangHai (abbreviate as SH), LiaoNing (abbreviate as LN), and JianSu (abbreviate as JS)—were selected. These games account for the final research dataset for this study. The four selected teams are objectively regarded as representing the top tier of women's softball in China today.

### 2.2 Variable selection

In this study, game outcomes—win (labeled as “1”) and loss (labeled as “0”)—were used as the target variables for the machine learning models. The input features for the models were derived from the ScorePAD system, which recorded detailed game statistics through post-game analysis and computation. Specifically, the data of the system were obtained from on-site records. The process of each inning was manually entered into the system, which then automatically generated all final statistical indicators. Partial data of all games can be accessed at http://www.softball.org.cn/. A total of 113 statistical indicators were initially collected. To enhance the model's efficiency, irrelevant variables, such as season summary statistics, player positions, jersey numbers, and player names, were removed, leaving 98 features that constituted the 98-dimensional feature space used for this study's dataset. As these features have different scales and units, normalization was performed to prevent scale differences from impacting model training.

Furthermore, to facilitate subsequent analysis and interpretation of game outcome predictions, the 98 features were categorized into three groups based on the offensive and defensive roles in softball: (1) Team Batting Totals: Indicators reflecting a team's batting performance; (2) Team Defense Totals: Indicators representing overall team defensive performance; (3) Pitching Totals: As pitchers play a distinct and critical role in defense, their statistics are classified separately to evaluate their impact on game outcomes. The features and classification results are detailed in Table 1. Additionally, all statistics collected from the ScorePAD system were recalculated to align with the seven-inning format of softball games. If an extra inning occurred, the actual number of innings was used to ensure the accuracy of the final statistics. The final dataset comprised 81 game samples, each represented by a 98-dimensional feature vector, denoted as $(D_{ij}{)}_{81\times 98}$.

**Table 1:** Feature classification and variable names.

Feature group	Indicators
Team batting totals	At-Bats (AB), Runs (R), Hits (H), Doubles (2B), Triples (3B), Home Runs (HR), Runs Batted In (RBI), Sacrifice Hits (SH), Sacrifice Flies (SF), Intentional Walks (IBB), Walks (BB), Strikeouts (K), Stolen Bases (SB), Caught Stealing (CS), Grounded into Double Plays (GDP), Batting Average (AVG), Bunt Foul 3rd Strikes (Kbf), Bunt Singles (BH), Catcher Interference (CINT), Caught Stealing 2nd (CS2), Caught Stealing 3rd (CS3), Caught Stealing Home (CSH), Fielder’s Choice (FC), Hits with 1 Out (1OutH), Hits with 2 Outs (2OutH), Hits with No Outs (NoOutH), Solo Home Runs (SoloHR), Infield Singles (IFH), Lineouts (LO), Obstruction (OBSTR), On-Base Percentage (OBP), On-base Plus Slugging Percentage (OPS), Plate Appearances (PA), Reach on Error (ERRCH), Runners Advanced (RA), Slugging Percentage (SLG), Stolen Base Percentage (SB%), Stolen On (STLON), Called Strikeouts (Kc), Hit by Pitch (HB), Batting Avg. with 2 Outs (2OutAvg), RBI with 2 Outs (2OutRBI), Stolen 2nd Base (STL2), Stolen 3rd Base (STL3), Hits with Runners in Scoring Position (RISPH), Batting Average with Runners in Scoring Position (RISP%)
Team Defense Totals	Total Chances (TC), Putouts (PO), Assists **(A)**, Errors (E), Double Plays (DP), Best Defensive Plays (BP), Errors—fielding (Ef), Errors—throwing (Et), Fielding Percentage (FLD%), Flyouts (FLYO), Foulouts (FOULO), Groundouts (GO), Passed Ball Percentage (PB%), Pickoffs (PKO), Pitches Received (PCHR), Total Chances (TC)
Pitching Totals	First Batter Average (P-FBAVG), First Batter Reach Percentage (P-FBR%), First Batter Reach (P-FBR), Walks (P-BB), Intentional Walks (P-IBB), Double Plays Induced (P-DPI), Doubles (P-2B), Earned Run (P-ER), First Pitch Strike Percentage (P-FPS%), First Pitch Strikes (P-FPS), Flyouts (P-FLYO), Groundouts (P-GO), Hits (P-H), Home Runs (P-HR), At-Bats Against (P-AP), Pitches-Balls (P-B), Pitches Put In-play (P-I), Pitches Behind in Count (P-BP), Pitches from Even Counts (P-EP), Pitches Fouled off (P-F), Pitches—Strikes (P-S), Total Pitches (P-TopP), Sacrifice Flies (P-SF), Sacrifice Hits (P-SH), Strikeout Percentage (P-K%), Strikeouts (P-K), Called Strikeouts (P-Kc), Strikeouts to Walks Percentage (P-KW%), Total At-Bats (P-TAB), Total Batters Faced (P-TBF), Triples (P-3B), Wild Pitches (P-WP), First Batter At-Bats (P-FBAB), First Batter Hits (P-FBH), Total First Batters Faced (P-FBF), and Innings Pitched (P-IP)

### 2.3 Model development and selection

As indicated by the features presented in Table 1, softball game data, comprising batting, defense, and pitching indicators, are inherently heterogeneous, including continuous and categorical data. This characteristic presents certain challenges for model development. Hence, data normalization was first applied. Considering the complexity of factors influencing softball game outcomes and the special nature of the collected data, this study selected Random Forest (RF), XGBoost, K-Nearest Neighbors (KNN), and Support Vector Machine (SVM) algorithms as candidate machine learning methods for game-outcome prediction. These algorithms were implemented using Python's scikit-learn library.

To enhance model generalizability, the sample dataset was randomly partitioned (without replacement) into a training data set and a testing data set following a 7:3 ratio, with the number of samples rounded to the nearest integer. The training data set was used for model training and parameter tuning, while the testing data set was reserved for performance evaluation. Supervised machine learning models based on RF, XGBoost, KNN, and SVM were developed to predict softball game outcomes. Model parameters were optimized through grid search cross-validation (GridSearchCV, CV = 5). Thus, optimal parameters were adaptively identified to minimize the loss function, as illustrated in (Equation 2):

$$\theta^{*}=\arg\;\min\xi (\theta )$$ (2)

where θ is a hyper-parameter of the model, ξ(θ) is the loss function.

Model performance was comprehensively evaluated using classic metrics from the machine learning domain, including AUC, F1-score, accuracy, precision, and recall. The model with the best performance was selected based on these metrics, while the confusion matrix and calibration were used to illustrate the performance of the selected model.

### 2.4 Experiment design

The collected game data first underwent preprocessing steps, including screening and data cleaning, which enabled the construction of the experimental dataset. Subsequently, the dataset was randomly partitioned (without replacement) into training and testing samples. Subsequently, supervised learning was performed separately for the four selected machine learning algorithms using the training and testing sets. Model results obtained from the testing data set were compared and analyzed based on the five evaluation metrics mentioned previously, and the best-performing model was identified. Finally, with the focus on the “win” scenario (target variable = 1), the selected model's predictions were explained, using the SHAP and PDP algorithms, from two perspectives: overall feature contributions and individual feature effects. These methods reveal how high-dimensional input features influence model predictions, along with the direction and magnitude of such influences, providing strategic references for customized in-game tactics for different teams.

### 2.5 Statistical methods

Statistical analysis was conducted on the experimental dataset used in this study. SPSS 26 software was employed to perform the Shapiro–Wilk test for normality. If the resulting *p*-value exceeded 0.05, indicating normal data distribution, descriptive statistics were presented as “mean ± standard deviation” $\left(\bar{x}\pm s\right)$. The paired-sample *t*-test was used to compare differences between the winning and losing groups. For data not following a normal distribution (*p* < 0.05), descriptive statistics were expressed using median and interquartile range (M [P25, P75]), and the Mann–Whitney *U* test was used to assess inter-group differences, with the significance set at *p* < 0.05. If an indicator showed a statistically significant difference under this criterion, the conclusion was that the indicator differed significantly between the winning and losing groups.

### 2.6 Team-level normalized SHAP calculation

To compare differences in feature importance across teams, the SHAP values were normalized at the team level. Using the trained model, we analyzed the top ten features ranked by their overall SHAP importance. This approach aimed to illustrate how the common key features identified by the overall model contribute specifically within samples from different teams. Considering that SHAP values can be either positive or negative, we used the absolute SHAP values to represent the magnitude of feature importance rather than the direction of influence. The top ten features were selected based on the highest mean absolute SHAP values calculated from the entire dataset using the trained model. The calculation of the mean absolute SHAP value at the team level is shown in (Equation 3):

$${\bar{S}}_{\;jt}=\frac{1}{n_{t}}\sum_{i=1}^{n_{t}}\left|s_{ijt}\right|$$ (3)

For team *t*, which has $n_{t}$ games and a feature set *j* ∈ {1, …, 10}, the SHAP value of the *j*-th feature in the *i*-th game is denoted as $s_{ijt}$. Subsequently, normalization was performed within each team to reflect the relative contribution proportion of each feature. The calculation formula is shown in (Equation 4):

$$\mathrm{Normalized}\;\mathrm{SHA}P_{\;jt}=\frac{{\bar{S}}_{\;jt}}{\sum_{k=1}^{10}{\bar{S}}_{kt}}\times 100\text{\%}$$ (4)

The normalized results were used to compare the relative contributions of the top ten key features across different teams, thereby revealing both the common and distinct winning factors among teams.

### 2.7 Estimation of 95% confidence intervals

To quantify the uncertainty of model predictions and performance metrics, 95% confidence intervals (CIs) were calculated using a Bootstrap resampling approach with 1,000 iterations. For RF and XGBoost, CIs for prediction probabilities were derived by resampling individual tree predictions, while for KNN and SVM, CIs were obtained by resampling the training set and retraining the models. Performance metrics were evaluated on the test set with Bootstrap resampling. At the team level, SHAP values of the top 10 features were bootstrapped within each team, and 95% CIs were calculated based on the distribution of mean absolute SHAP values after normalization.

## 3 Results

### 3.1 Results of independent samples tests

The evaluation results comparing the statistical indicators between the winning and losing teams are shown in Table 2. The dataset comprises indicators from winning and losing teams across games. That is, AB, R, H, 2B, RBI, SH, BB, IBB, GDP, AVG, 1OutH, 2OutH, NoOutH, OBP, OPS, PA, ERRCH, SLG, HB, 2OutAvg, 2OutRBI, RISPH, RISP%, PO, E, Ef, FLD%, PCHR, P-FBAVG, P-FBR%, P-FBR, P-BB, P-DPI, P-2B, P-ER, P-FPS, P-H, P-HR, P-B, P-I, P-BP, P-S, P-TotP, P-SH, P-K%, P-K, P-KW%, P-TAB, P-TBF, P-FBH, P-FBF, P-IP. As shown, all indicator differences are statistically significant (*p* < 0.05), clearly demonstrating the appropriateness and representativeness of the selected dataset.

**Table 2:** Comparisons of game indicators between winning and losing teams.

Variables	AB $\left(\bar{x}\pm s\right)$	R [*M*(*P*_25_, *P*_75_)]	H [*M*(*P*_25_, *P*_75_)]	2B [*M*(*P*_25_, *P*_75_)]	3B [*M*(*P*_25_, *P*_75_)]	HR [*M*(*P*_25_, *P*_75_)]	RBI [*M*(*P*_25_, *P*_75_)]	SH [*M*(*P*_25_, *P*_75_)]
Wins (1)	25.7 ± 4.4	7 (5, 9)	9 (7, 10)	2 (1, 3)	0 (0, 1)	0 (0, 1)	6 (4, 8)	1 (1, 2)
Losses (0)	23.5 ± 5.1	2 (1, 5)	5 (3, 7)	1 (0, 2)	0 (0, 0)	0 (0, 0)	2 (1, 3)	1 (0, 1)
Test statistic value	2.272^a^	8.558^b^	6.591^b^	3.999^b^	1.715^b^	2.782^b^	8.347^b^	4.166^b^
*p*-value	0.003*	<0.001*	<0.001*	<0.001*	0.086	0.05	<0.001*	<0.001*
ES	0.33	1.24	0.93	0.40	0.10	0.22	1.25	0.52
95% CI	(0.01, 0.64)	(0.90, 1.58)	(0.60, 1.26)	(0.08, 0.71)	(−0.21, 0.41)	(−0.09, 0.53)	(0.91, 1.59)	(0.21, 0.84)
Variables	SF [*M*(*P*_25_, *P*_75_)]	BB [*M*(*P*_25_, *P*_75_)]	IBB [*M*(*P*_25_, *P*_75_)]	K [*M*(*P*_25_, *P*_75_)]	SB [*M*(*P*_25_, *P*_75_)]	CS [*M*(*P*_25_, *P*_75_)]	GDP [*M*(*P*_25_, *P*_75_)]	AVG $\left(\bar{x}\pm s\right)$
Wins (1)	0 (0, 0)	2 (2, 4)	0 (0, 1)	3 (2, 5.5)	1 (0, 2)	0 (0, 0)	0 (0, 0)	0.355 ± 0.119
Losses (0)	0 (0, 0)	2 (1, 3)	0 (0, 0)	5 (3, 7)	1 (0, 2)	0 (0, 1)	0 (0, 0)	0.215 ± 0.099
Test statistic value	1.913^b^	2.445^b^	1.115^b^	−2.788^b^	1.822^b^	−0.652^b^	−2.874^b^	0.140^a^
*p*-value	0.056	0.015*	0.265	0.005	0.068	0.514	0.004*	<0.001*
ES	0.06	0.24	−0.01	−0.58	0.26	−0.04	−0.16	1.01
95% CI	(−0.25, 0.37)	(−0.07, 0.55)	(−0.33, 0.29)	(−0.89, −0.26)	(−0.05, 0.57)	(−0.36, 0.26)	(−0.47, 0.15)	(0.72, 1.38)
Variables	Kbf [*M*(*P*_25_, *P*_75_)]	BH [*M*(*P*_25_, *P*_75_)]	CINT [*M*(*P*_25_, *P*_75_)]	CS2 [*M*(*P*_25_, *P*_75_)]	CS3 [*M*(*P*_25_, *P*_75_)]	CSH [*M*(*P*_25_, *P*_75_)]	FC [*M*(*P*_25_, *P*_75_)]	1OutH [*M*(*P*_25_, *P*_75_)]
Wins (1)	0 (0, 0)	0 (0, 0.5)	0 (0, 0)	0 (0, 0)	0 (0, 0)	0 (0, 0)	1 (0, 1)	3 (2, 4)
Losses (0)	0 (0, 0)	0 (0, 0)	0 (0, 0)	0 (0, 0)	0 (0, 0)	0 (0, 0)	1 (0, 2)	1.5 (1, 2)
Test statistic value	0^b^	0.997^b^	−1.000^b^	−0.081^b^	−0.464^b^	0^b^	0.303^b^	5.063^b^
*p*-value	1	0.319	0.317	0.935	0.643	1.000	0.762	<0.001*
ES	/	0.05	0	−0.09	0.16	/	−0.05	0.57
95% CI	/	(−0.26, 0.37)	/	(−0.40, 0.22)	(−0.15, 0.50)	/	(−0.36, 0.27)	(0.25, 0.89)
Variables	2OutH [*M*(*P*_25_, *P*_75_)]	NoOutH [*M*(*P*_25_, *P*_75_)]	SoloHR [*M*(*P*_25_, *P*_75_)]	IFH [*M*(*P*_25_, *P*_75_)]	LO [*M*(*P*_25_, *P*_75_)]	OBP $\left(\bar{x}\pm s\right)$	OBSTR [*M*(*P*_25_, *P*_75_)]	PA $\left(\bar{x}\pm s\right)$
Wins (1)	3 (1.5, 4)	3 (1, 4)	0 (0, 0)	1 (0, 2)	0 (0, 1)	0.428 ± 0.114	0 (0, 0)	31.2 ± 4.9
Losses (0)	1 (1, 2)	2 (1, 3)	0 (0, 0)	1 (1, 1)	1 (0, 1)	0.288 ± 0.102	0 (0, 0)	26.9 ± 6.8
Test statistic value	4.196^b^	2.856^b^	0.767^b^	1.648^b^	−1.367^b^	0.140^a^	0^b^	4.160^a^
*p*-value	<0.001*	0.004*	0.443	0.099	0.172	<0.001*	1	<0.001*
ES	0.53	0.48	0.09	0.17	0	1.10	/	0.52
95% CI	(0.21, 0.85)	(0.16, 0.79)	(−0.22, 0.40)	(−0.13, 0.49)	(−0.13, 0.13)	(0.76, 1.43)	/	(0.21, 0.84)
Variables	OPS [*M*(*P*_25_, *P*_75_)]	ERRCH [*M*(*P*_25_, *P*_75_)]	RA [*M*(*P*_25_, *P*_75_)]	SB% [*M*(*P*_25_, *P*_75_)]	STLON [*M*(*P*_25_, *P*_75_)]	Kc [*M*(*P*_25_, *P*_75_)]	HB [*M*(*P*_25_, *P*_75_)]	
Wins (1)	0.955 (0.751, 1.178)	1 (0, 1)	0 (0, 0)	1.000 (0, 1.000)	1 (0, 1.5)	1 (0, 2)	1 (0, 1)	
Losses (0)	0.591 (0.411, 0.785)	0 (0, 1)	0 (0, 0)	0.584 (0, 1.000)	1 (0, 2)	1 (0, 2)	0 (0, 1)	
Test statistic value	6.998^b^	2.494^b^	−1.000^b^	1.631^b^	−1.822^b^	−0.614^b^	1.990^b^	
*p*-value	<0.001*	0.013*	0.317	0.103	0.068	0.539	0.047*	
ES	0.90	0.21	0	0.23	−0.31	−0.25	0.41	
95% CI	(0.58, 1.23)	(−0.11, 0.52)	/	(−0.08, 0.55)	(−0.62, 0.01)	(−0.57, 0.06)	(0.09, 0.72)	
Variables	SLG [*M*(*P*_25_, *P*_75_)]	2OutAvg [*M*(*P*_25_, *P*_75_)]	2OutRBI [*M*(*P*_25_, *P*_75_)]	STL2 [*M*(*P*_25_, *P*_75_)]	STL3 [*M*(*P*_25_, *P*_75_)]	RISPH [*M*(*P*_25_, *P*_75_)]	PO [*M*(*P*_25_, *P*_75_)]	
Wins (1)	0.500 (0.361, 0.667)	0.333 (0.200, 0.400)	2 (1, 3)	1 (0, 1)	0 (0, 0)	4 (3, 5)	21 (16.5, 21)	
Losses (0)	0.306 (0.188, 0.440)	0.200 (0.125, 0.300)	1 (0, 2)	1 (0, 2)	0 (0, 0)	1 (0, 2)	18 (15, 21)	
Test statistic value	6.635^b^	4.590^b^	5.422^b^	1.187^b^	1.707^b^	7.917^b^	2.799^b^	
*p*-value	<0.001*	<0.001*	<0.001*	0.235	0.088	<0.001*	0.005*	
ES	0.75	0.60	0.66	0.14	0.37	1.14	0.52	
95% CI	(0.43, 1.08)	(0.29, 0.92)	(0.35, 0.98)	(−0.17, 0.45)	(0.05, 0.68)	(0.80, 1.47)	(0.20, 0.835)	
Variables	RISP% [*M*(*P*_25_, *P*_75_)]	A [*M*(*P*_25_, *P*_75_)]	E [*M*(*P*_25_, *P*_75_)]	DP [*M*(*P*_25_, *P*_75_)]	BP [*M*(*P*_25_, *P*_75_)]	Ef [*M*(*P*_25_, *P*_75_)]	Et [*M*(*P*_25_, *P*_75_)]	
Wins (1)	0.364 (0.273, 0.486)	7 (6, 9)	1 (0, 1)	0 (0, 0.5)	0 (0, 0)	0 (0, 1)	0 (0, 1)	
Losses (0)	0.191 (0, 0.273)	7 (6, 10)	1 (1, 2)	0 (0, 0)	0 (0, 0)	1 (0, 1)	0 (0, 1)	
Test statistic value	6.439^b^	−0.759^b^	−3.884^b^	1.611^b^	0.020^b^	−3.467^b^	−1.335^b^	
*p*-value	<0.001*	0.448	<0.001*	0.107	0.984	0.001*	0.176	
ES	0.80	−0.09	−0.45	0.08	0.13	−0.46	−0.14	
95% CI	(0.48, 1.12)	(−0.41, 0.21)	(−0.76, −0.13)	(−0.23, 0.39)	(−0.18, 0.44)	(−0.78, −0.15)	(−0.45, 0.17)	
Variables	FLD% [*M*(*P*_25_, *P*_75_)]	FLYO [*M*(*P*_25_, *P*_75_)]	FOULO [*M*(*P*_25_, *P*_75_)]	GO [*M*(*P*_25_, *P*_75_)]	PB% [*M*(*P*_25_, *P*_75_)]	PKO [*M*(*P*_25_, *P*_75_)]	PCHR $\left(\bar{x}\pm s\right)$	
Wins (1)	0.981 (0.964, 1)	4 (2, 5)	0 (0, 1)	5 (4, 7)	0 (0, 0)	0 (0, 0)	99.0 ± 26.0	
Losses (0)	0.963 (0.923, 0.971)	4 (2, 6)	0 (0, 1)	5 (4, 7)	0 (0, 0)	0 (0, 0)	109.9 ± 25.3	
Test statistic value	4.195^b^	−0.509^b^	0.162^b^	0.250^b^	0.219^b^	−1.000^b^	−11.025^a^	
*p*-value	<0.001*	0.611	0.872	0.802	0.827	0.317	0.007*	
ES	0.53	−0.04	0.03	0.11	−0.18	0	−0.42	
95% CI	(0.22, 0.85)	(−0.35, 0.27)	(−0.28, 0.34)	(−0.20.0.42)	(−0.50, 0.13)	/	(−0.73, −0.10)	
Variables	TC $\left(\bar{x}\pm s\right)$	P-FBAVG [*M*(*P*_25_, *P*_75_)]	P-FBR% [*M*(*P*_25_, *P*_75_)]	P-FBR [*M*(*P*_25_, *P*_75_)]	P-BB [*M*(*P*_25_, *P*_75_)]	P-IBB [*M*(*P*_25_, *P*_75_)]	P-DPI [*M*(*P*_25_, *P*_75_)]	
Wins (1)	27.2 ± 6.1	0 (0, 0)	0 (0, 0.333)	0 (0, 1)	2 (1, 3)	0 (0, 0)	0 (0, 0)	
Losses (0)	26.8 ± 6.34	0 (0, 0.5)	0.417 (0, 0.750)	1 (0, 2)	2 (1, 4)	0 (0, 1)	0 (0, 0)	
Test statistic value	0.444^a^	−3.791^b^	−3.953^b^	−4.543^b^	−2.481^b^	−1.161^b^	2.892^b^	
*p*-value	0.651	<0.001*	<0.001*	<0.001*	0.013*	0.246	0.004*	
ES	0.17	−0.64	−0.52	−0.73	−0.23	0.06	0.16	
95% CI	(−0.14, 0.48)	(−0.96, −0.32)	(−0.84, −0.21)	(1.05, −0.41)	(−0.54, 0.09)	(−0.25, 0.37)	(−0.15, 0.472)	
Variables	P-2B [*M*(*P*_25_, *P*_75_)]	P-ER [*M*(*P*_25_, *P*_75_)]	P-FBS% $\left(\bar{x}\pm s\right)$	P-FPS $\left(\bar{x}\pm s\right)$	P-FLYO [*M*(*P*_25_, *P*_75_)]	P-GO [*M*(*P*_25_, *P*_75_)]	P-H [*M*(*P*_25_, *P*_75_)]	
Wins (1)	1 (0, 2)	2 (0, 3)	0.562 ± 0.101	15.1 ± 4.0	6 (4, 9)	7 (5, 9)	5 (3, 7)	
Losses (0)	2 (1, 3)	6 (4, 8)	0.535 ± 0.098	16.7 ± 4.0	5 (4, 7)	7.5 (6, 10)	9 (6, 11)	
Test statistic value	−3.974^b^	−8.077^b^	0.027^a^	−1.605^a^	0.969^b^	−1.055^b^	−6.601^b^	
*p*-value	<0.001*	<0.001*	0.087	0.012*	0.332	0.291	<0.001*	
ES	−0.38	−1.21	0.33	−0.18	0.05	−0.19	−0.97	
95% CI	(−0.69, −0.06)	(−1.55, −0.89)	(0.02, 0.64)	(−0.50, −0.13)	(−0.26, 0.36)	(−0.50, 0.12)	(−1.30, −0.64)	
Variables	P-HR [*M*(*P*_25_, *P*_75_)]	P-AP $\left(\bar{x}\pm s\right)$	P-B [*M*(*P*_25_, *P*_75_)]	P-I [*M*(*P*_25_, *P*_75_)]	P-BP [*M*(*P*_25_, *P*_75_)]	P-EP $\left(\bar{x}\pm s\right)$	P-F [*M*(*P*_25_, *P*_75_)]	P-S $\left(\bar{x}\pm s\right)$
Wins (1)	0 (0, 0)	33.2 ± 11.6	34 (29, 44)	20 (16, 24)	26 (21, 35)	17.0 ± 5.7	17 (13, 20)	61.4 ± 14.7
Losses (0)	0 (0, 1)	33.7 ± 12.3	45 (35, 53)	23 (21, 26)	36 (27, 44)	17.6 ± 5.4	17 (14, 21)	67.0 ± 14.0
Test statistic value	−2.720^b^	−0.556^a^	−3.983^b^	−4.630^b^	−4.250^b^	−0.679^a^	−1.265^b^	−5.593^a^
*p*-value	0.007*	0.768	<0.001*	<0.001*	<0.001*	0.438	0.206	0.014*
ES	−0.21	0.26	−0.52	−0.71	−0.65	−0.18	−0.02	−0.20
95% CI	(−0.52, 0.10)	(−0.05, 0.57)	(−0.84, −0.21)	(−1.03, −0.39)	(−0.97, −0.33)	(−0.49, 0.13)	(−0.33, 0.29)	(−0.51, 0.11)
Variables	P-TotP [*M*(*P*_25_, *P*_75_)]	P-SF [*M*(*P*_25_, *P*_75_)]	P-SH [*M*(*P*_25_, *P*_75_)]	P-K% [*M*(*P*_25_, *P*_75_)]	P-K [*M*(*P*_25_, *P*_75_)]	P-Kc [*M*(*P*_25_, *P*_75_)]	P-KW% [*M*(*P*_25_, *P*_75_)]	
Wins (1)	98 (82.5, 116)	0 (0, 0)	1 (0, 1)	0.179 (0.103, 0.262)	5 (2.5, 7)	1 (0, 2)	1.5 (0.5, 2.75)	
Losses (0)	110 (95, 128)	0 (0, 0)	1 (1, 2)	0.110 (0.063, 0.161)	3 (2, 6)	1 (0, 2)	1.0 (0.3, 2.0)	
Test statistic value	−3.086^b^	−1.911^b^	−3.907^b^	3.987^b^	2.758^b^	0.605^b^	1.975^b^	
p-value	0.002*	0.056	<0.001*	<0.001*	0.006*	0.545	0.048*	
ES	−0.04	−0.06	−0.54	0.62	0.59	0.23	0.50	
95%C I	(−0.35, 0.27)	(−0.37, 0.25)	(−0.86, −0.22)	(0.30, 0.94)	(0.28, 0.91)	(−0.08, 0.54)	(0.19, 0.82)	
Variables	P-TAB $\left(\bar{x}\pm s\right)$	P-TBF $\left(\bar{x}\pm s\right)$	P-3B [*M*(*P*_25_, *P*_75_)]	P-WP [*M*(*P*_25_, *P*_75_)]	P-FBAB [*M*(*P*_25_, *P*_75_)]	P-FBH [*M*(*P*_25_, *P*_75_)]	P-FBF [*M*(*P*_25_, *P*_75_)]	P-IP [*M*(*P*_25_, *P*_75_)]
Wins (1)	23.6 ± 5.3	26.8 ± 7.2	0 (0, 0.5)	0 (0, 0.5)	1 (1, 2)	0 (0, 0)	1 (1, 2)	7 (5.5, 7)
Losses (0)	25.8 ± 4.4	31.2 ± 4.9	0 (0, 1)	0 (0, 1)	2 (1, 2)	0 (0, 1)	2 (1, 3)	6 (5, 7)
Test statistic value	−2.210^a^	−4.370^a^	−1.715^b^	−1.478^b^	−1.845^b^	−3.840^b^	−3.000^b^	2.639^b^
*p*-value	0.004*	<0.001*	0.086	0.139	0.065	<0.001*	0.003*	0.008*
ES	−0.30	−0.50	−0.12	−0.42	−0.42	−0.69	−0.45	0.49
95% CI	(−0.61, 0.01)	(−0.82, −0.19)	(−0.43, 0.19)	(−0.74, −0.11)	(−0.74, −0.11)	(−1.01, −0.37)	(−0.76, −0.13)	(0.17, 0.80)

a Represents the *t*-value

b Represents the *U*-value. *Indicates significant differences in the feature.

### 3.2 Model training and selection

**Figure 2:**
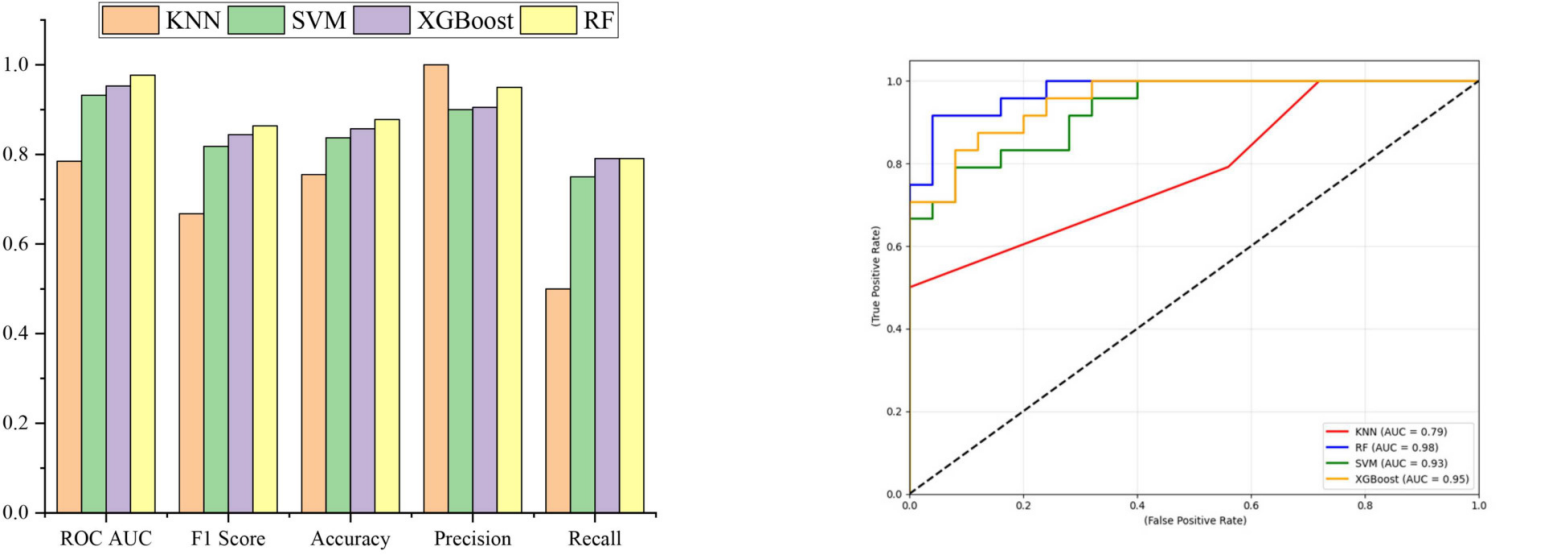
Evaluation metrics of 4 models. **(a)** Bar chart of model evaluation metrics. **(b)** Roc curves for identifying “Win” in the test sets of four models.

Table 3 presents the evaluation metrics for the four machine-learning models, which are based on the testing dataset described in Section 1.3. Corresponding performance indicators are visualized in Figure 2a, and the ROC curves illustrating the win-loss predictions of each of the four models are presented in Figure 2b. Figure 2a demonstrates that, excluding the KNN model, all the models exhibit strong generalization capabilities on the test dataset. A comprehensive assessment of Table 3 and Figures 2a–b shows that the RF model obtained the highest ROC AUC and F1 scores among the compared models, implying comparatively better predictive performance and generalization within this dataset.

**Table 3:** Performance of 4 models.

Models	ROC AUC	F1 Score	Accuracy	Precision	Recall	AUC 95%CI
KNN	0.785	0.688	0.755	1	0.500	(0.690,0.920)
SVM	0.932	0.818	0.837	0.900	0.750	(0.838,0.983)
XGboost	0.953	0.844	0.837	0.905	0.791	(0.901,0.989)
RF	0.977	0.864	0.878	0.950	0.791	(0.938,0.993)

This superior performance is primarily attributed to the RF being an ensemble learning method based on decision trees. It constructs multiple decision trees and combines their outputs, enhancing predictive accuracy and stability. Given a training dataset $=\{\left(x_{i},y_{i}\right){\}}_{i=1}^{N}$, where $x_{i}$ represents input features and $y_{i}$ denotes corresponding labels, RF employs Bootstrap Sampling to select multiple subsets randomly from the training dataset to train *M* decision trees $f_{m}(x)$. The final prediction is obtained through an ensemble strategy. For classification tasks, the majority voting method is applied, as shown in (Equation 5):

$$\hat{y}=\arg\;\max\overset{M}{\underset{m=1}{\sum }}\;\psi (\;f_{m}(x)=c)$$ (5)

where ψ(⋅) is an indicator function, and *c* represents the class labels. For regression tasks, a simple averaging approach is used, as shown in (Equation 6):

$$\hat{y}=\frac{1}{M}\overset{M}{\underset{m=1}{\sum }}\;f_{m}(x)$$ (6)

Additionally, to further enhance generalization, RF introduces feature subset selection at each split during the construction of each tree. Specifically, at each node, only subset *k* of the total *d* features (k≤d) is randomly selected for optimal splitting, as illustrated by (Equation 7):

$$k=\lfloor \log_{2}d+1\rfloor$$ (7)

**Figure 3:**
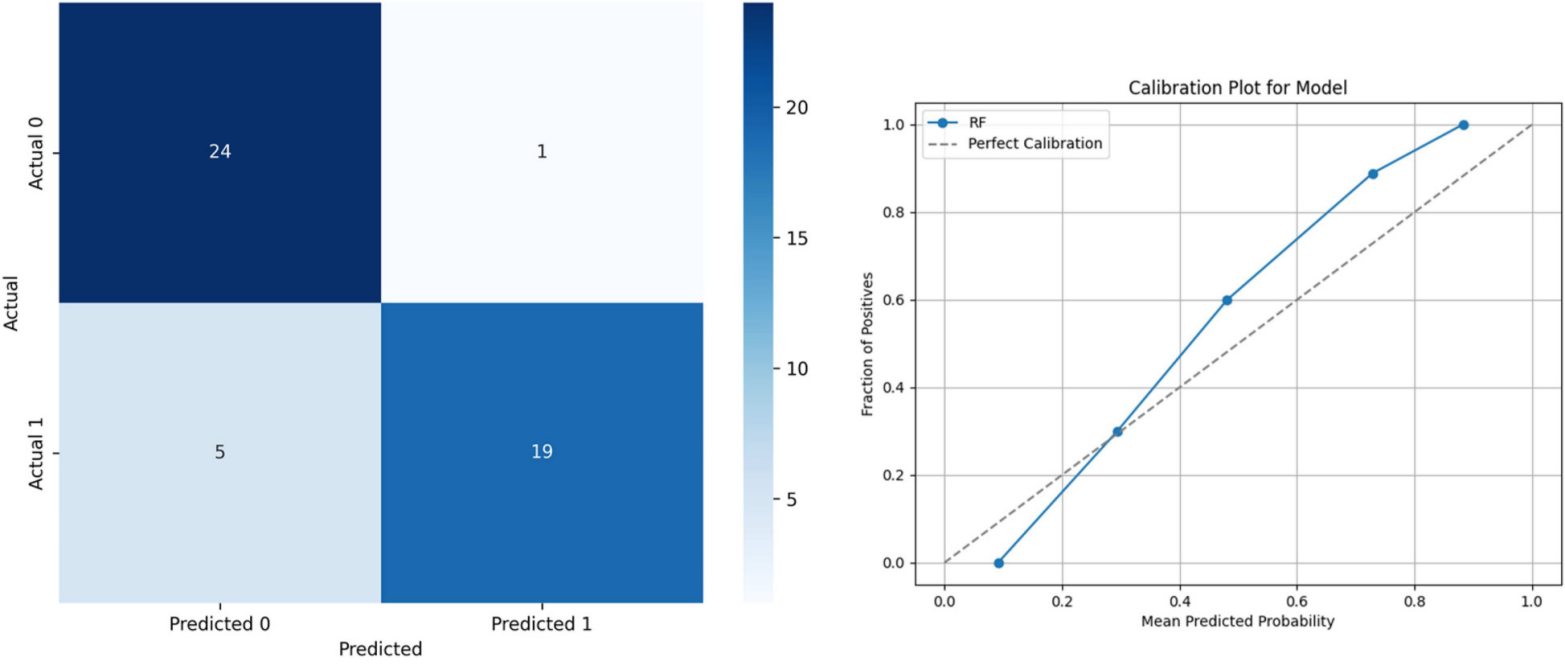
The performance of the selected model. **(a)** Confusion Matrix. **(b)** Calibration.

This method effectively reduces correlations between individual decision trees, enhancing overall model stability. Consequently, RF demonstrates strong predictive performance for game outcome predictions. The key hyperparameters optimized in this study include max_depth = 5, n_estimators = 100, min_samples_split = 5 and min_samples_leaf = 2. The other hyper-parameters were configured at their default values. Given these advantages, the outputs from the RF model were chosen as inputs for subsequent SHAP and PDP analyses in this study. The performance of the selected model is shown in Figure 3.

### 3.3 Identification of game outcome–associated factors using the SHAP algorithm

Based on the RF prediction model described in Section 2.2, the SHAP algorithm was used to calculate the Shapley values for each feature in the dataset $(D_{ij}{)}_{81\times 98}$, quantifying their respective contributions to the RF model's game outcome predictions. For a model with *n* input features, the Shapley value $\phi_{i}$ of feature *i* is calculated using cooperative game theory, as shown in (Equation 8):

$$\phi_{i}=\sum_{S\subset N,i\in S}\frac{(|S|-1)!(n-|S|)!}{n!}[v(S)-v(S-\{i\})]$$ (8)

where *N* represents the set of all features, S is a subset containing a portion of these features, |S| denotes the size (number of elements) of subset *S*, and v(S) is the model prediction corresponding to feature subset *S*.

**Figure 4:**
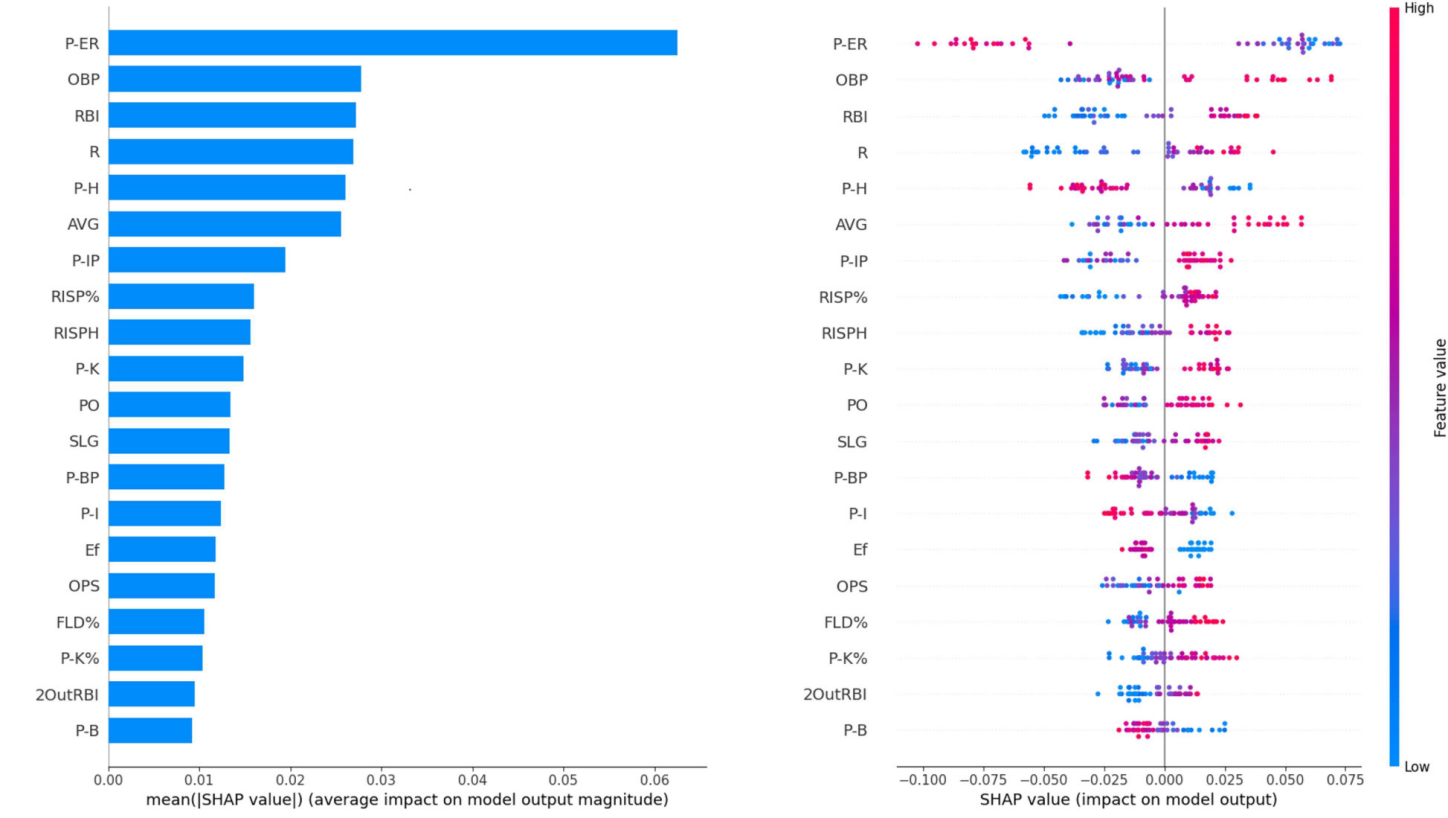
The Shapley value of features. **(a)** The average Shapley value of features. **(b)** The global feature importance.

The top ten features ranked by Shapley value through comparative calculations include P-ER, OBP, RBI, R, P-H, AVG, P-IP, RISP%, RISPH, P-K. Figure 4a presents the mean Shapley values for all features, while Figure 4b shows the top 20 features ranked by Shapley value across the dataset. Given the large number (98) of total features, Figure 4b is termed the “Global Feature Importance” plot for convenience. In Figure 4b, each row on the *y*-axis represents an individual feature, while the *x*-axis indicates the magnitude of the Shapley values. A larger Shapley value signifies greater contribution to the predictive outcome. Positive Shapley values imply a positive impact on the predicted outcome, whereas negative values indicate the opposite. The color of each dot corresponds to the value of a feature instance, transitioning from blue (low feature values) to red (high feature values). As illustrated in Figure 4b, features such as OBP, RBI, R, AVG, P-IP, RISP%, RISPH, P-K, PO, SLG, OPS, FLD%, P-K%, 2OutRBI have red dots clustered toward the right side of the *x*-axis, with blue dots clustered toward the left side. This pattern suggests that as the values of these features increase, they positively influence game outcome prediction. Conversely, for features like P-ER, P-H, P-BP, P-I, Ef, P-B, blue dots are clustered toward the right, while red dots are clustered toward the left—lower values for these features negatively impact game outcome prediction (i.e., predicting win or loss).

### 3.4 Feature explanation using PDP

#### 3.4.1 One-Dimensional PDP analysis results

Using the top ten features identified by SHAP analysis in Section 2.3, including P-ER and OBP, we further employed the PDP algorithm to analyze how individual features influenced the predicted game outcomes. For one-dimensional PDP analysis, given the model f(x) described in (Equation 4), where *x* is a feature vector, the PDP effect of feature $j^{\mathrm{th}}$ is calculated according to (Equation 9):

$$\mathrm{PD}P_{j}(x_{j})=\frac{1}{n}\sum_{i=1}^{n}\;f(x_{ij},x_{-i})$$ (9)

where $x_{ij}$ represents the value of feature $j^{\mathrm{th}}$ in sample *i*, while $x_{-i}$ denotes all other features in sample *i* except for feature $j^{\mathrm{th}}$.

**Figure 5:**
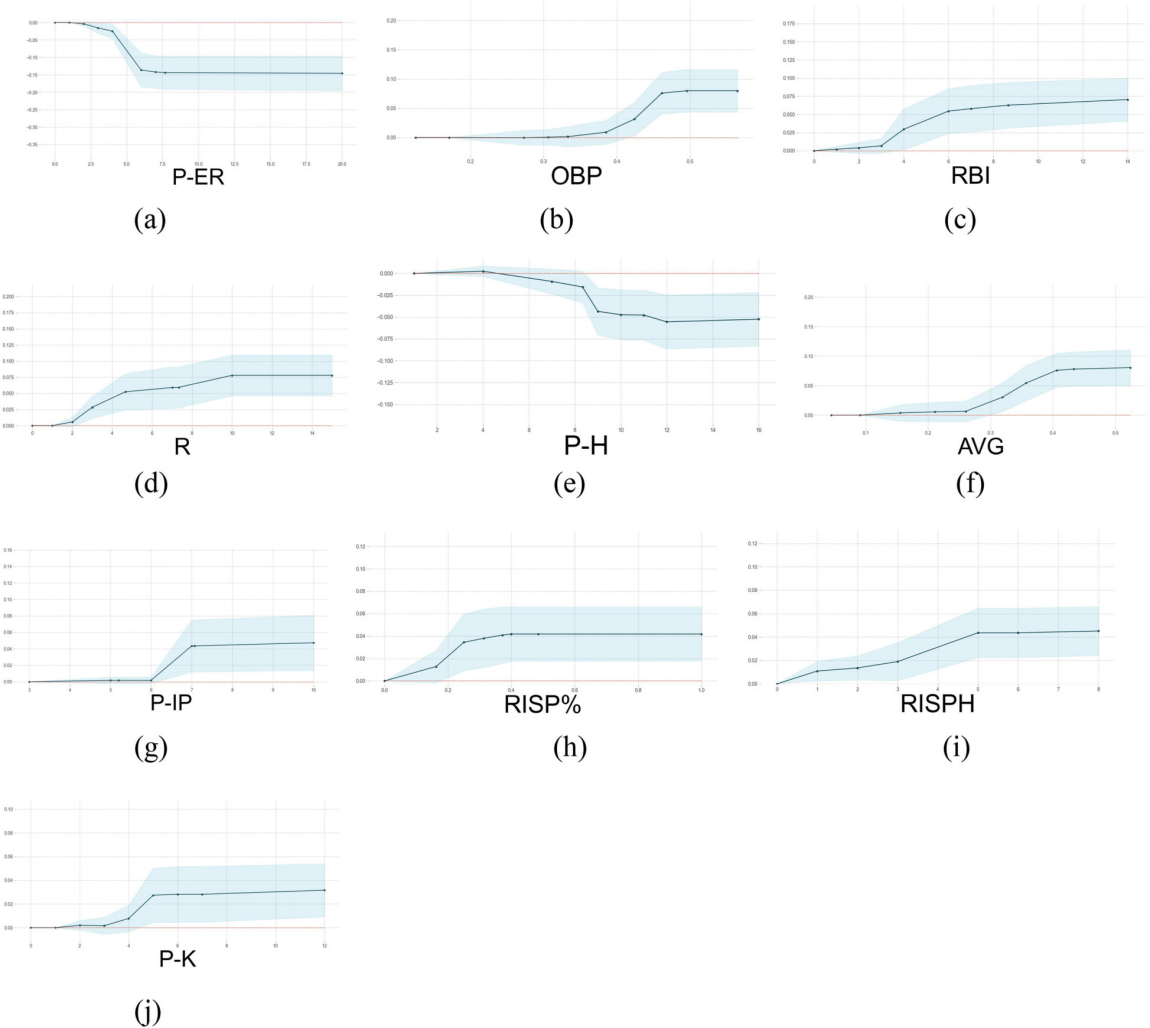
The 1-D PDP of the top ten features ranked by the Shapley value. The *X*-axis represents the selected feature (count or percentage), and the *Y*-axis represents the predicted probability of winning at each corresponding feature value. **(a–j)** displays the partial dependence plots (PDPs) for the top 10 features based on SHAP values.

Figure 5 presents the PDP analysis results, with all analyses based on the dataset $(D_{ij}{)}_{81\times 98}$ used in this study.

#### 3.4.2 Two-dimensional PDP analysis results

**Figure 6:**
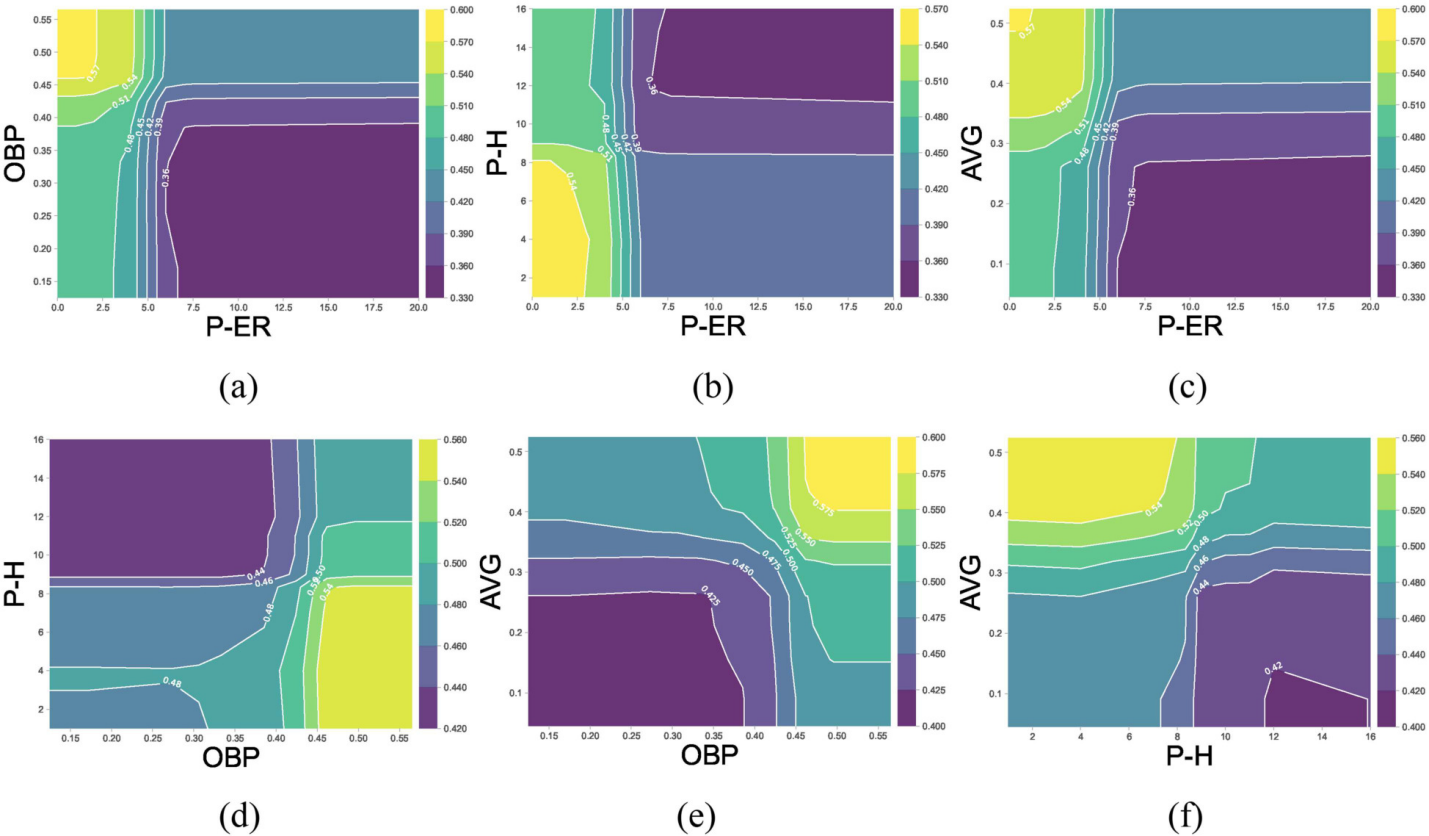
The 2-D PDP interaction effects of the P-RE, OBP, P-H, and AVG. The *X*-axis and *Y*-axis represent the selected feature (count or percentage), *Z*-axis (represented by color) predicted win probability under the interaction effects of the selected variables. **(a–f)** shows the 2D PDP interaction plots for several key variables.

To further examine the interaction effects among important features contributing to game outcomes, this study analyzed the top six features ranked by Shapley value. Since Runs Batted In (RBI) and Runs Scored (R) have direct and dominant effects on game results, this section focuses on the interaction effects of other potential key features. Therefore, the remaining four features—Earned Runs (P-ER), On-Base Percentage (OBP), Hits (P-H), and Batting Average (AVG)—were selected for two-dimensional PDP analysis. The results are illustrated in Figure 6; the *X*-axis and *Y*-axis represent the values of the two interacting features, while the *Z*-axis, visualized through contour shading, represents their combined effect on winning probability. Lighter colors correspond to higher winning probabilities, while darker colors indicate lower winning probabilities. When the *Z*-axis value exceeds 0.5, the interaction effect of the selected feature pair has a significant positive contribution to the game outcome (i.e., increasing the probability of winning). Figures 6a–f provides an intuitive visualization of how selected feature interactions influence game results, offering quantitative insights into decisive factors for further strategic analysis.

### 3.5 Analysis of decisive factors for sample teams

**Figure 7:**
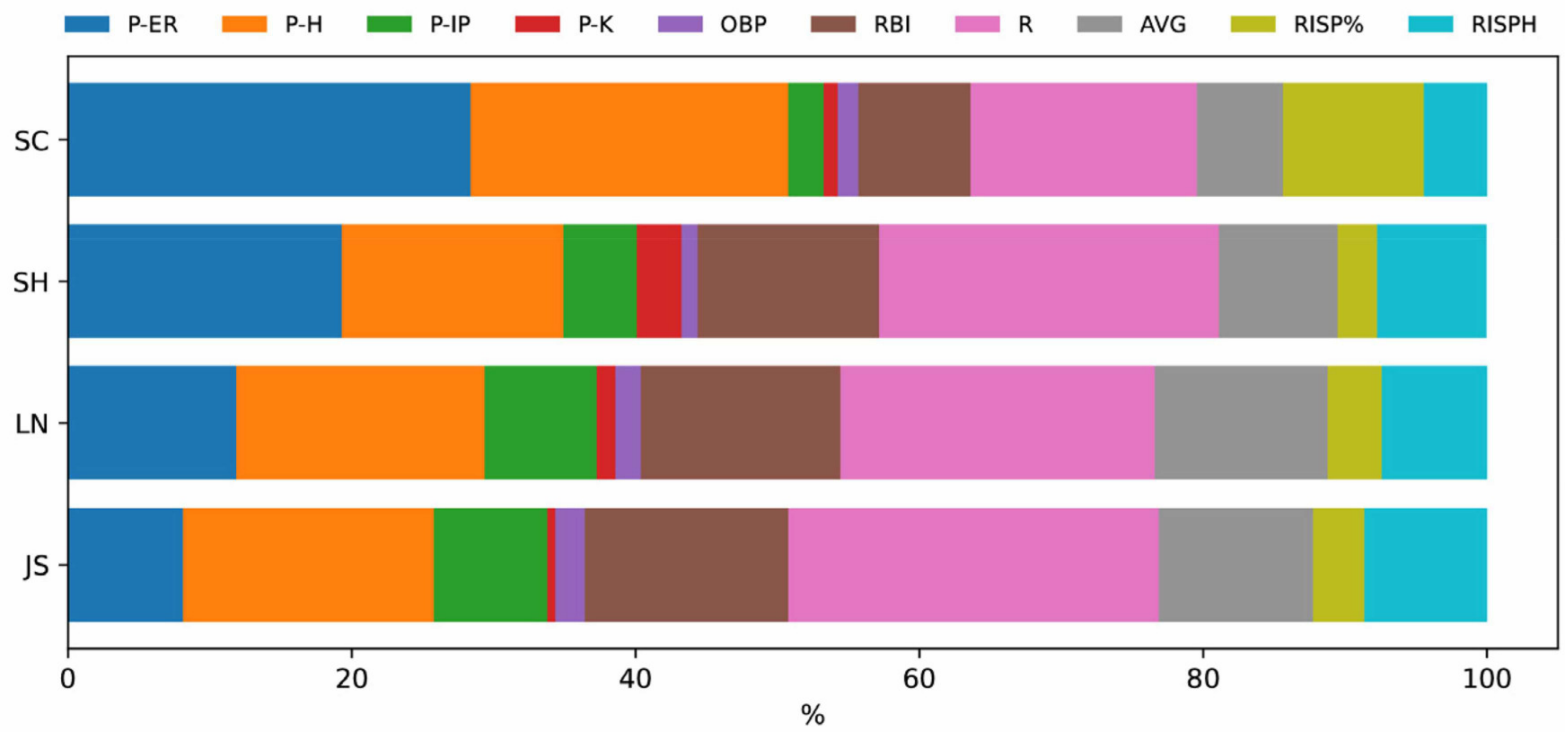
Normalized contribution values of Key decisive factors for each team.

Table 4 and Figure 7 illustrate the contributions of the decisive factors identified by the RF model for the four sample teams (SC, *n* = 21; SH, *n* = 16; LN, *n* = 23; JS, *n* = 21). The results reveal distinct strategic differences among teams in terms of the relative importance of different factors affecting game outcomes. SC relies heavily on pitching performance, with pitching indicators contributing 54.26% to their overall key factors. The most influential factors for SC are Earned Runs (P-ER) and Hits (P-H)—SC's winning strategy focuses on limiting opponents' scoring and maintaining control over the game through strong pitching performance. The batting totals of teams SH, LN, and JS are more important contributing factors. These three teams place higher emphasis on offensive performance, with offensive indicators contributing 56.76%, 61.38%, and 65.63% to their decisive factors, respectively. In particular, Runs Batted In (RBI) and Batting Average (AVG) stand out as the most significant offensive contributors for these teams. This highlights the importance of scoring ability and batting efficiency in their winning strategies. LN and JS also rely heavily on Batting Average with Runners in Scoring Position (RISP%)—these teams prioritize capitalizing on key offensive opportunities to maximize scoring potential.

**Table 4:** Shapley values (absolute) of decisive factors for team victories.

Indicators	SC (95%CI)	SH (95%CI)	LN (95%CI)	JS (95%CI)
P-ER	0.0195 (0.0173, 0.0229)	0.0100 (0.0083, 0.0117)	0.0059 (0.0043, 0.0067)	0.0040 (0.0028, 0.0053)
P-H	0.0154 (0.0121, 0.0178)	0.0081 (0.0073, 0.0089)	0.0087 (0.0083, 0.0091)	0.0088 (0.0063, 0.0108)
P-IP	0.0017 (0.0001, 0.0031)	0.0027 (0.0025, 0.0030)	0.0039 (0, 0.0077)	0.0040 (0.0017, 0.0063)
P-K	0.0006 (0, 0.0010)	0.0016 (0.0009, 0.0022)	0.0007 (0, 0.0015)	0.0003 (0, 0.0005)
OBP	0.0010 (0.0003, 0.0018)	0.0006 (0.0004, 0.0008)	0.0009 (0.0005, 0.0014)	0.0010 (0.0002, 0.0019)
RBI	0.0054 (0.0034, 0.0079)	0.0066 (0.0063, 0.0070)	0.0070 (0.0059, 0.0083)	0.0071 (0.0063, 0.0078)
R	0.0110 (0.0093, 0.0122)	0.0125 (0.0083, 0.0168)	0.0110 (0.0099, 0.0123)	0.0130 (0.0099, 0.0164)
AVG	0.0041 (0.0033, 0.0048)	0.0044 (0.0034, 0.0055)	0.0060 (0.0044, 0.0078)	0.0054 (0.0043, 0.0066)
RISP%	0.0068 (0.0031, 0.0092)	0.0015 (0.0009, 0.0019)	0.0019 (0.0011, 0.0029)	0.0018 (0.0011, 0.0025)
RISPH	0.0007 (0.0004, 0.0011)	0.0040 (0.0033, 0.0047)	0.0037 (0.0033, 0.0042)	0.0043 (0.0037, 0.0048)
Pitching Metrics	54.2566%	43.2400%	38.6154%	34.3655%
Offensive Metrics	45.7434%	56.7600%	61.3846%	65.6345%
Total	100%	100%	100%	100%

## 4 Discussion

This study developed machine learning models to investigate the key factors influencing game outcomes and employed SHAP and PDP algorithms to identify variables that significantly contribute to winning or losing. The findings enhance the understanding of performance determinants and offer potential guidance for data-driven decision-making in competitive contexts.

### 4.1 Analysis of key factors

As shown in Table 2 and Figure 4, the top ten features (i.e., factors) ranked by Shapley value exhibit statistically significant differences between winning and losing teams (*p* < 0.05, Table 2) and have a positive impacts on the outcome variable. The winning teams generally have higher mean or median values for positive key indicators compared to the losing teams, and vice versa. To further examine pitching and batting indicators, we analyze the impact of five key metrics in pitching (P-ER and P-H) and batting (OBP, AVG and SLG)). Among pitching indicators, Pitcher's Earned Runs (P-ER) was identified as the most influential winning factor in this study. This finding aligns with previous NCAA Division I softball analytics, where P-ER is widely used as a core metric for evaluating pitcher performance (22). P-ER represents runs allowed directly because of pitching performance, such as runs scored after a walk or hits leading to runners advancing. A low P-ER indicates that the pitcher effectively suppresses opposing batters, making it difficult for them to make solid contact. Teams with lower P-ER typically have stronger pitching performance. Pitcher's Hits Allowed (P-H) is a key factor contributing to P-ER. Figures 6d,f indicate that when a pitcher allows a certain number of hits, the team's offensive ability (AVG > 30% or OBP > 40%) must compensate for the defensive shortcomings to maintain a chance of winning. The data from this study indicate that when P-H exceeds 11, the team's probability of winning decreases significantly. Figure 6b shows that P-ER and P-H exhibit a strong negative interaction effect with offensive metrics, suggesting that pitching performance is crucial in determining the outcome of a game. Among batting indicators, On-Base Percentage (OBP) and Batting Average (AVG) ranked 2nd and 5th, respectively, in terms of winning impact. Hakes et al. (18) found that OBP and Slugging Percentage (SLG) are key differentiators of winning probability in Major League Baseball (MLB), as they are highly correlated with Runs (R). This study further reveals that OBP contributes approximately twice as much to winning probability as SLG. Interestingly, AVG ranks higher than SLG in terms of Shapley values—AVG has a greater impact on winning in softball when compared to SLG. This difference can be attributed to variations between baseball and softball in terms of game dynamics, including field dimensions and tactical priorities. Smaller softball fields result in less time for outfielders to react, restricting base advancement on extra-base hits and making multi-base hits less common. Softball strategies emphasize short-ball tactics, focusing on bunting and aggressive baserunning to create scoring opportunities. Shorter base paths (18.3 m in softball) encourage a single-hit, station-to-station offensive approach to generate runs. These factors explain why Batting Average (AVG) has a more significant impact on winning probability in softball than Slugging Percentage (SLG). Moreover, Runs Batted In (RBI), Hits with Runners in Scoring Position (RISPH), and Batting Average with Runners in Scoring Position (RISP%) ranked 3rd, 8th, and 9th, respectively, in terms of winning impact. These offensive statistics are more directly related to scoring than OBP and AVG. RBI serves as a crucial measure of a player's contribution to team scoring. A high RBI value indicates that a batter successfully capitalizes on scoring opportunities, increasing team offensive efficiency. RISPH and RISP% measure a team's ability to convert scoring opportunities into runs. Higher RISPH and RISP% values indicate that the team efficiently capitalizes on scoring opportunities by driving runners home when possible. This significantly boosts winning probability.

In summary, this section highlights the pivotal roles of both pitching and batting performance—particularly metrics such as P-ER, OBP, and AVG—in influencing game outcomes, and underscores the importance of effective run prevention and timely hitting in maximizing a team's winning probability.

### 4.2 Discussion on customized winning strategies of different teams

The findings obtained from Figures 4–7 and Table 4 reveal significant differences in pitching- and batting-focused strategies based on the top ten key indicators. This section further analyzes the customized winning strategies of the four teams respectively. SC relies heavily on pitching performance, with pitching indicators contributing 54.26% to their overall key factors (Table 4). P-ER and P-H are the most influential factors in SC's strategy. This indicates that SC prioritizes controlling opponents' scoring to maintain an advantage, emphasizing the pitcher's central role in game strategy. Their approach maximizes game control by minimizing runs allowed. In contrast, SH, LN, and JS place higher emphasis on batting performance, with offensive indicators contributing 56.76%, 61.38%, and 65.63%, respectively, to their overall key factors (Table 4). The core offensive key factors for these teams are RBI and AVG—these teams focus on enhancing scoring ability and batting performance to increase their likelihood of winning. Such an offense-driven strategy enables these teams to gain an advantage through high-efficiency offensive play, regardless of game scenarios. Further analysis reveals that Batting Average with Runners in Scoring Position (RISP%) plays a particularly significant role in LN's and JS's success. This suggests that LN and JS prioritize scoring in key offensive situations, emphasizing clutch hitting in high-pressure moments to maintain a competitive edge. The strong impact of RISP% also highlights these teams' focus on executing under pressure, which requires advanced tactical skills and strong mental resilience.

In summary, SC, SH, LN, and JS exhibit distinct dependencies on pitching and offensive performance. However, regardless of the primary winning strategy, RBI and AVG consistently emerge as critical factors across all teams. This finding reinforces that scoring ability and stable batting performance remain core determinants of victory, regardless of whether a team prioritizes offense or pitching. Cairney (20) found that in MLB, the contribution ratio of offensive and defensive abilities to winning probability is approximately 1:1. Similarly, this study suggests that a balanced approach between offense and defense is crucial for overall team performance and resilience. JS and LN, as traditional domestic powerhouses, dominate offensively in national competitions. Nevertheless, against teams with no significant weaknesses in either pitching or offense, such as the USA and Japan, their reliance on offense may not be sufficient. When facing such elite opponents, dominant pitching performances can neutralize strong offenses, making it difficult for JS and LN to generate runs. Hence, this study recommends that the Chinese women's softball teams also focus on strengthening their pitching depth.

## 5 Conclusion and future outlook

### 5.1 Conclusion

This study developed a RF-based model to investigate the key factors influencing game outcomes and utilized SHAP and PDP algorithms to analyze the explainability of the model. Based on this approach, a systematic analysis was conducted to identify key factors influencing game outcomes and to explore interactions among different features. First, the SHAP explainability analysis revealed that batting and pitching indicators are crucial in determining game outcomes. Among these indicators, Pitcher's Earned Runs (P-ER) demonstrated the highest importance and explanatory power, while other metrics, such as On-Base Percentage (OBP), Pitcher's Hits Allowed (P-H), and Batting Average (AVG), also contributed significantly to predicting game results. Second, the two-dimensional PDP analysis demonstrated that P-ER and P-H are strong negative-effect indicators—an increase in either metric substantially reduces the probability of winning. In particular, excessive earned runs or hits allowed by a pitcher could significantly reduce a team's likelihood of securing victory. Finally, this study identified team-specific differences in winning strategies. While SC relies primarily on pitching performance, SH, LN, and JS adopt offense-dominant strategies.

### 5.2 Limitations and future directions

Although this study successfully mined decisive factors using machine learning models and explainability techniques and revealed notable differences in pitching and offensive strategies among the four sample teams, several limitations remain. First, although machine learning excels in handling complex nonlinear relationships, feature selection remains critical in determining the accuracy and effectiveness of predictions. In MLB, new performance metrics have been continuously introduced in recent years to evaluate batting and pitching performance. For instance, emerging batting metrics include Batting Average on Balls in Play (BABIP), Weighted On-Base Average (wOBA), and Expected Weighted On-Base Average (xwOBA). In pitching, commonly used indicators include Fielding Independent Pitching (FIP) and Adjusted Earned Run Average (ERA). Owing to limitations of the current ScorePAD system, this study could not incorporate these metrics, presenting a constraint in feature engineering. Second, the dataset used in this study was primarily drawn from Chinese softball teams, with a relatively limited sample size. This restricts the generalizability of the findings. Moving forward, expanding the dataset to include international competitions and long-term game records will be crucial for improving the applicability of the research findings. Performing a global-scale study with multi-year data will be a key focus of future research. Finally, although the indicators with high SHAP values indeed represent quantitative process descriptions that determine game outcomes in the real world, caution is still required when applying model-derived features to practical contexts. In addition, the PDP assumes independence among features, and given the correlations between some variables, its interpretation should be approached carefully. Further validation using accumulated local effects (ALE) or conditional analyses is recommended.

## Data Availability

The raw data supporting the conclusions of this article will be made available by the authors, without undue reservation.
